# Supplementary material for: Physiological and socioeconomic characteristics predict COVID-19 mortality and resource utilization in Brazil
Source: PLoS One. 2020 Oct 14;15(10):e0240346. doi: 10.1371/journal.pone.0240346 (PMC7556459; doi:10.1371/journal.pone.0240346)
Supplement: S1 File — (ZIP) [file pone.0240346.s001.zip › plos_one_supp/supporting_info.pdf]

Physiological and socioeconomic characteristics predict COVID-19 mortality and resource utilization in Brazil Supplementary Material

Salomón Wollenstein-Betech [1,2], Amanda A. B. Silva [4], Julia L. Fleck [4], Christos G. Cassandras [1,2], Ioannis Ch. Paschalidis [1,2,3,*],

1 Division of Systems Engineering, Boston University, Boston, MA, USA

2 Department of Electrical and Computer Engineering, Boston University, Boston, MA, USA

3 Department of Biomedical Engineering, Boston University, Boston, MA, USA

4 Department of Industrial Engineering, Pontifícia Universidade Católica do Rio de Janeiro, RJ, Brazil

[*]Corresponding author
E-mail: yannisp@bu.edu (IP)

Supporting information

Ventilator coefficients, ORs and CIs

Table 1. Mechanical ventilator need coefficients for ℓ_2-LR.

	β	CI (2.5)	CI (97.5)	OR	CI (2.5)	CI (97.5)
Region_South	-1.122	-1.221	-1.023	0.326	0.295	0.360
Region_Southeast	-1.111	-1.158	-1.064	0.329	0.314	0.345
Region_Midwest	-0.796	-0.898	-0.693	0.451	0.407	0.500
Cough	-0.637	-0.678	-0.595	0.529	0.508	0.552
Region_Northeast	-0.631	-0.683	-0.579	0.532	0.505	0.561
Obesity	0.631	0.546	0.716	1.880	1.726	2.047
SpO2 less 95%	0.561	0.519	0.603	1.752	1.680	1.828
Age 0-30	-0.543	-0.639	-0.446	0.581	0.528	0.640
Throat	-0.441	-0.493	-0.389	0.643	0.611	0.678
Fever	-0.385	-0.425	-0.345	0.681	0.654	0.709
Xray Thorax Result Consolidation	0.365	0.265	0.464	1.440	1.304	1.590
Schooling Superior	-0.345	-0.438	-0.252	0.708	0.645	0.778
Diarrhea	-0.335	-0.396	-0.275	0.715	0.673	0.759
Gender_F	-0.317	-0.354	-0.281	0.728	0.702	0.755
Antiviral Use	0.303	0.264	0.343	1.355	1.302	1.409
Other Symptoms	-0.303	-0.343	-0.263	0.739	0.710	0.769
Liver Chronic Disease	0.287	0.116	0.457	1.332	1.123	1.580
Another Chronic Pneumopathy	0.282	0.196	0.369	1.326	1.216	1.446
Renal Chronic Disease	0.275	0.195	0.356	1.317	1.215	1.427
Schooling Medium 1-3	-0.264	-0.328	-0.200	0.768	0.721	0.819
Other Risks	0.199	0.159	0.239	1.220	1.172	1.270
Vomiting	-0.181	-0.257	-0.104	0.835	0.773	0.901
Hematologic Disease	0.172	-0.005	0.349	1.187	0.995	1.417
Cardiovascular Disease	0.166	0.126	0.205	1.180	1.135	1.228
Respiratory Discomfort	0.165	0.124	0.207	1.180	1.132	1.230
Contracted At Hospital	0.159	0.047	0.270	1.172	1.049	1.311
Diabetes	0.157	0.115	0.198	1.170	1.122	1.219
Immunosuppression	0.148	0.048	0.249	1.160	1.049	1.283
Race White	-0.054	-0.099	-0.009	0.948	0.906	0.991
Hospital Public	-0.046	-0.091	-0.000	0.955	0.913	1.000
Dyspnea	0.026	-0.017	0.070	1.027	0.983	1.073
Hospital Private	-0.020	-0.066	0.026	0.980	0.937	1.026
Acute Respiratory Distress Syndrome	0.019	-0.022	0.061	1.020	0.978	1.063
Neurological Disease	0.014	-0.074	0.102	1.014	0.929	1.108

Results from *advanced* models

In addition to two models presented in the main body of the paper, we train *advanced* models for predicting the events of interest (mortality and use of ventilation). These *advanced* models are provided with more information about the evolution of the disease. For mortality, we include information on whether a patient is in an ICU and on a ventilator. When this data is provided, the accuracy and AUC of the model increase by 9.7% and 9.6%, respectively, compared to the one presented in Table 2 and Fig. 3. Conversely, for the ventilation model, we include the variable ICU which improves the

accuracy and AUC of the model by 10.3% and 12.6% respectively. The performance metrics of the *advanced* mortality and ventilator models are in Tables 2 and 3, respectively, and in Figures 1a and 1b we present the ORs and CIs for the two *advanced* models.

Table 2. Mortality results

	SVM-l1	LR-l1	LR-l2	RF	XGBoost
Accuracy	0.768	0.768	0.768	0.767	0.774
F1w	0.767	0.768	0.767	0.767	0.773
AUC	0.853	0.853	0.853	0.850	0.856

Table 3. Ventilator results

	SVM-l1	LR-l1	LR-l2	RF	XGBoost
Accuracy	0.832	0.833	0.832	0.834	0.832
F1w	0.835	0.834	0.834	0.833	0.831
AUC	0.865	0.865	0.865	0.867	0.864

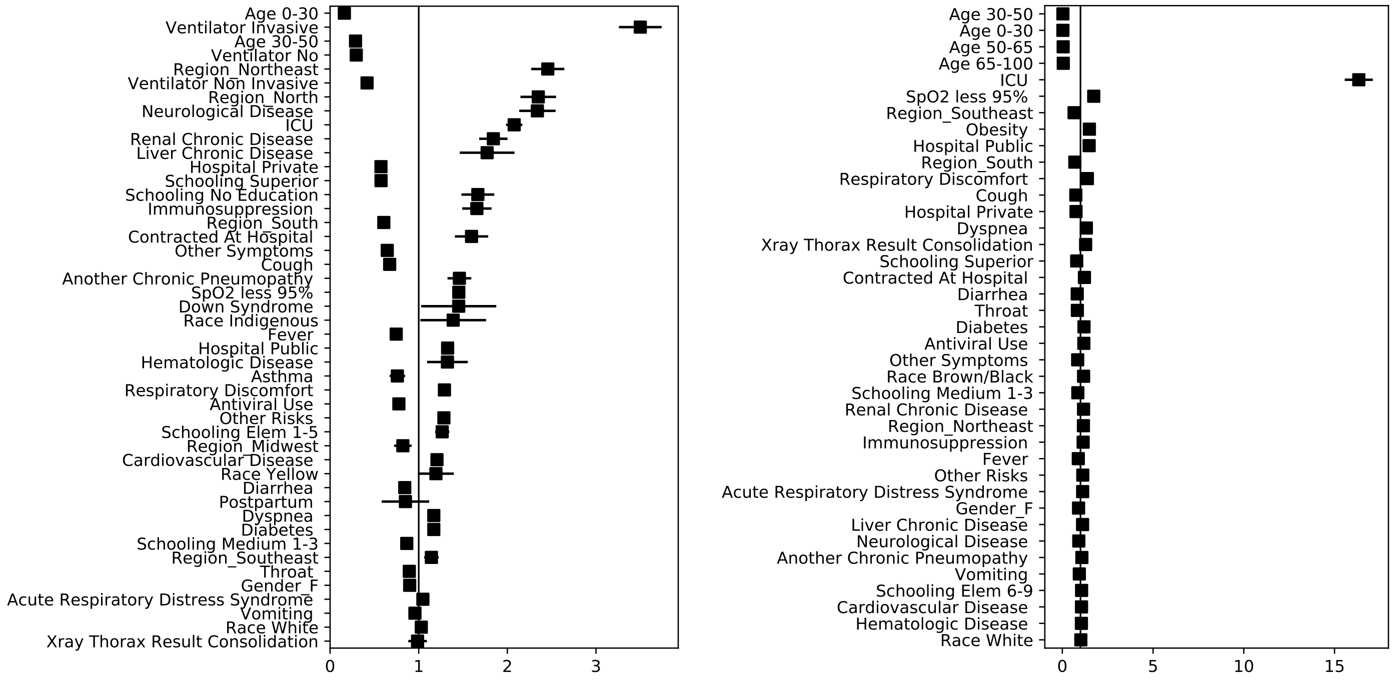

(a) ℓ_2−LR, Mortality.

(b) ℓ_2−LR, Ventilator.

Fig 1. Odds ratio and confidence intervals.

Classification

To build the predictive models, we assume we are given a vector (denoted by bold letters) of features $\mathbf{x}_i \in \mathrm{R}^D$, and the outcome $y_i = \{-1, 1\}$ of each patient $i = 1, \ldots, n$. We use D to denote the number of features observed for each patient.

Sparse linear support vector machines

A support vector machine (SVM) is a binary classifier that consists of a hyperplane on the feature space that separates the data into two classes (half-spaces) [1]. Its main idea is to select a hyperplane such that the distance between the closest point in each class to the hyperplane is maximized. In cases when the data is not linearly separable, meaning that there is no hyperplane able to perfectly separate all points, the so-called soft-margin SVM is employed which tolerates and penalize this misclassification, its formulation is:

$$\begin{aligned}
\min_{\beta_0,\boldsymbol{\beta},\boldsymbol{\xi}_i} \quad & \frac{1}{2}\|\boldsymbol{\beta}\|^2 + C\sum_{i=1}^{n}\xi_i + \rho\|\boldsymbol{\beta}\|_1 \\
\text{s.t.} \quad & \xi_i \geq 0, \quad \forall \in 1,\ldots,n, \\
& y_i(\mathbf{x}_i'\boldsymbol{\beta} + \beta_0) \geq 1 - \xi_i, \quad \forall i \in 1,\ldots,n,
\end{aligned}$$

where the hyperplane is characterized by the normal vector and intercept $(\boldsymbol{\beta}, \beta_0)$, and C is the misclassification penalty. This formulation differs from a standard SVM by the use of an ℓ_1-norm regularizer, inspired by robustness arguments [2], which induces sparsity to model. The scalar ρ represents the strength of the regularizer. This problem can be reformulated as a convex quadratic programming problem which can be solved using standard solvers.

Sparse logistic regression

Comparable to sparse SVM, logistic regression (LR) [25] is also an interpretable binary linear classifier. This model estimates the posterior probability of an outcome y_i (now represented as $\{0, 1\}$) as a function of a linear combination of the features $\mathbf{x}_i$. To achieve that, it uses parameters $\boldsymbol{\theta}$ that weight the input features and an offset θ_0. To select $(\boldsymbol{\theta}, \theta_0)$, the method maximizes the log-likelihood of the training data by applying gradient-based algorithms. In particular, LR has been highly relevant in the medical literature because it predicts the probability that a sample belongs to the positive (or negative) class, as well as, it yields easy to interpret results. In this work, we employ a sparse (ℓ_1-regularized) LR which includes an additional term in the objective function

proportional to $\|\boldsymbol{\theta}\|_1$. Similar as in sparse SVM, we include this term to provide robustness to the model. The training optimization problem is:

$$\min_{\boldsymbol{\theta},\theta_0} \quad \sum_{i=1}^{n}(-\log p(y_i|\mathbf{x}_i;\boldsymbol{\theta},\beta_0)) + \lambda\|\boldsymbol{\beta}\|_1,$$

where $p(y_i = 1|\mathbf{x}_i;\boldsymbol{\theta},\theta_0) = 1/(1+e^{(-\theta_0-\boldsymbol{\theta}'\mathbf{x}_i)}) = 1 - p(y=-1|\mathbf{x}_i;\boldsymbol{\theta},\theta_0)$, and λ is controlling the sparsity term. Similarly, an ℓ_2-regularized LR uses the same formulation as above but replacing $\|\boldsymbol{\beta}\|_1$ with $\|\boldsymbol{\beta}\|_2$. Note that for $\lambda = 0$, we have the standard logistic regression model.

Random forests

A Random Forest (RF) [3] often produce among the most accurate models for binary classification. These models are part of a larger set of predictors called ensemble methods whose main idea is to reduce the variance of a prediction. To achieve this variance reduction, this technique trains many noisy, but approximately unbiased, classifiers and predicts a new sample based on the majority vote of these weak classifiers. RF are ensemble of decision trees (DT), which are grown using data obtained through random sampling with replacement from the training set. A DT is *fully grown* until a minimum size (or depth) is reached. Theory has showm that even if the outcome of a single DT is noisy due to the sampling process, the average of many DTs is not, as long as these trees are not highly correlated. A critical disadvantage of ensemble methods is its lack for interpretability. This due to the fact that every prediction is obtained by majority voting of many (hundreds or thousands) weak and small DTs.

XGBoost

Recently, ensemble methods have been shown to be the best in class for binary classification tasks using structured data. XGBoost (which stands for Extreme Gradient Boosting) [4] is a decision-tree-based ensemble model that uses a gradient boosting framework. In fact, this model, has been credited with winning numerous Kaggle competitions and has being used widely in cutting-edge industry applications. However, as any ensemble method, it lacks interpretability.

References

1. Cortes C, Vapnik V. Support-vector networks. Machine Learning. 1995;20:273–297.
2. Chen R, Paschalidis IC. A Robust Learning Approach for Regression Models Based on Distributionally Robust Optimization. Journal of Machine Learning Research. 2018;19(13).
3. Breiman L. Random forests. Machine learning. 2001;45(1):5–32.
4. Chen T, Guestrin C. XGBoost: A Scalable Tree Boosting System. In: Proceedings of the 22nd ACM SIGKDD International Conference on Knowledge Discovery and Data Mining. KDD '16. San Francisco, California, USA: Association for Computing Machinery; 2016. p. 785–794. Available from: https://doi.org/10.1145/2939672.2939785.
