# Supplementary figures and images for: Physiological and socioeconomic characteristics predict COVID-19 mortality and resource utilization in Brazil
Source: PLoS One. 2020 Oct 14;15(10):e0240346. doi: 10.1371/journal.pone.0240346 (PMC7556459; doi:10.1371/journal.pone.0240346)

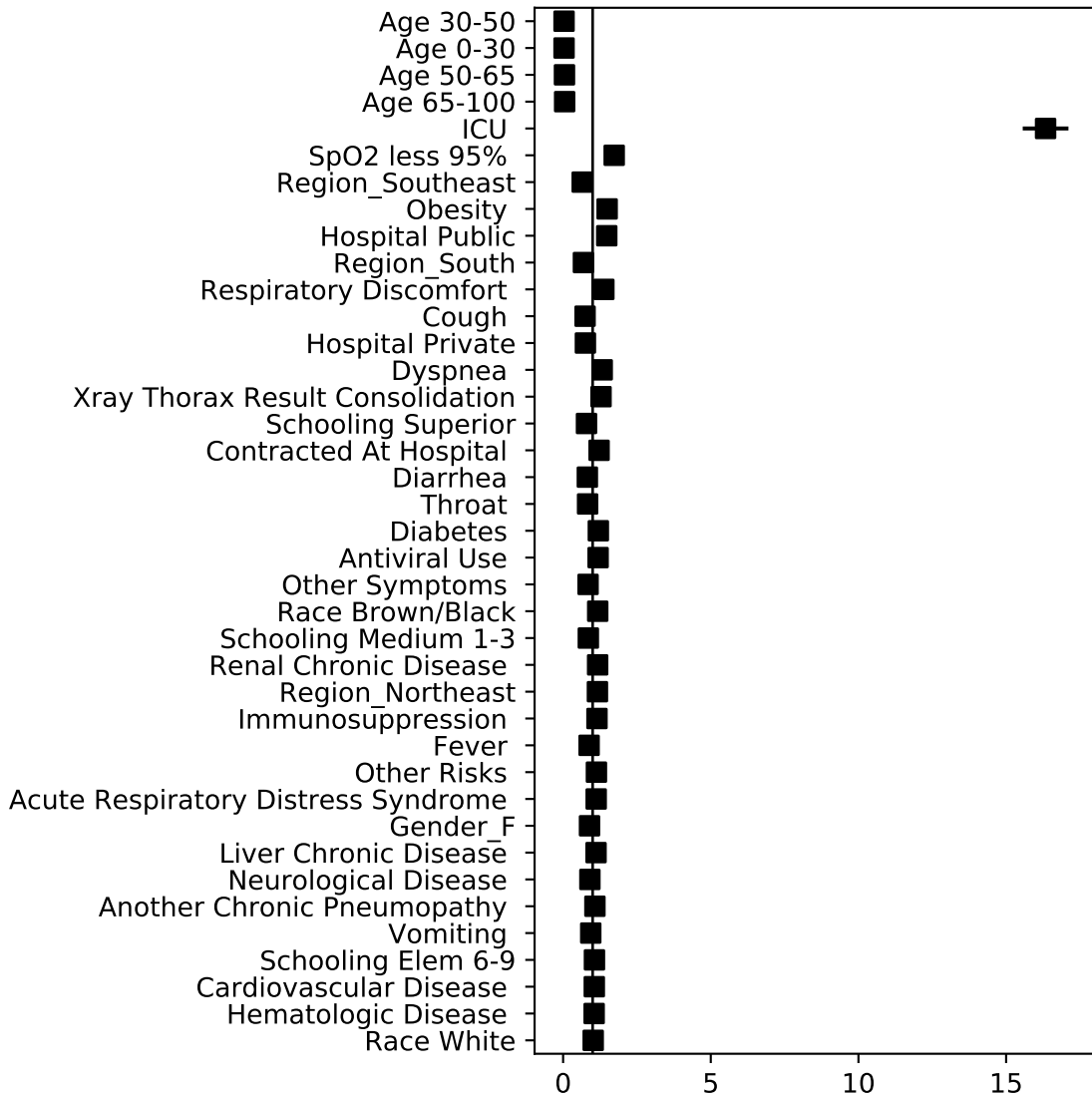

Supplement: S1 File — (ZIP) [file pone.0240346.s001.zip › plos_one_supp/figs/Ventilator_w_ICU_vars.pdf]

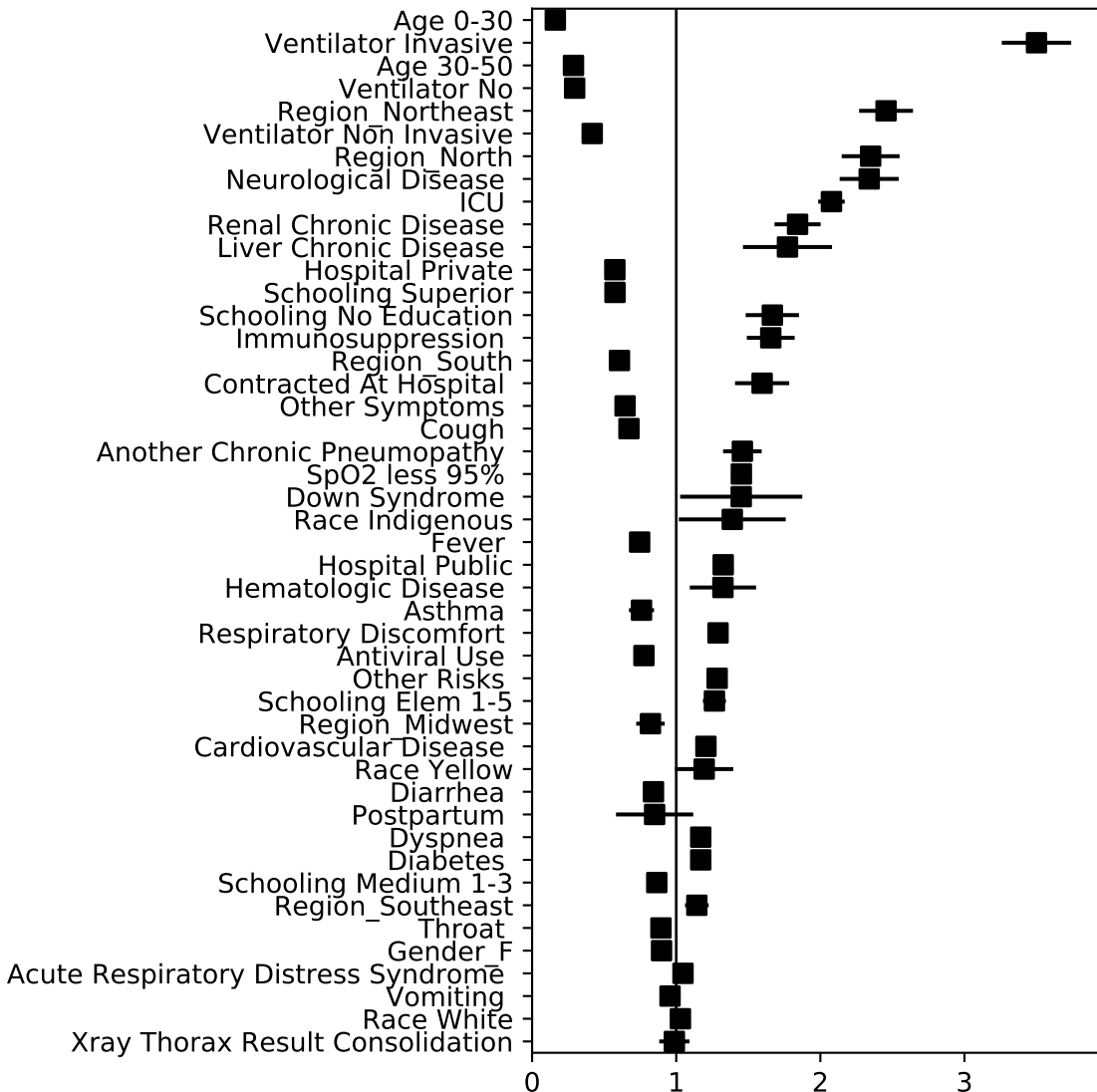

Supplement: S1 File — (ZIP) [file pone.0240346.s001.zip › plos_one_supp/figs/Death_1_vars.pdf]
