# Supplementary material for: Physiological and socioeconomic characteristics predict COVID-19 mortality and resource utilization in Brazil
Source: PLoS One. 2020 Oct 14;15(10):e0240346. doi: 10.1371/journal.pone.0240346 (PMC7556459; doi:10.1371/journal.pone.0240346)
Supplement: Attachment — Submitted filename: Rebuttal PLOS ONE.docx [file pone.0240346.s002.docx]

**Response to Reviews**

We appreciate the time and effort that the editor and the reviewers dedicated to providing feedback on our manuscript and we are grateful for their insightful. We have addressed all of the journal requirements and reviewer comments (significant modifications in the manuscript are shown in blue text to make it easier to read these changes).

Based on the reviewers comments, the most important revisions are the following (and are reflected with blue in the revised paper):

- Updated numbers of cases worldwide and in Brazil.
- Clarification of the values in Table 1.
- We include relevant information from the tables in the text of the Results section.
- We discuss the coefficient of cardiovascular disease and we support our results with references to previous related work.
- Included in the Discussion section a practical perspective of this work.
- Referenced all Figures and Tables.
- Stylistic changes in results and discussion sections.

Following are point-by-point responses to each of the reviewers' comments and Journal Requirements.

**Journal Requirements:**

1. Please ensure that your manuscript meets PLOS ONE's style requirements, including those for file naming. The PLOS ONE style templates can be found at

We have made changes to our manuscript to fulfill PLOS ONE’s style requirements.

2. Thank you for including your ethics statement: "The study analyzed publicly available data that have been fully de-identified, so it is not considered human subject research." Please revise this to state "The study analyzed publicly available data that have been fully de-identified, so additional ethical approval was not required”.

We have changed the ethics statement to the one provided.

3. Please include the date(s) on which you accessed the databases or records to obtain the data used in your study.

We have included the date on which we accessed the dataset in the manuscript.

4. For studies involving humans categorized by race/ethnicity, age, disease/disabilities, religion, sex/gender, sexual orientation, or other socially constructed groupings, authors should:

1) Explicitly describe their methods of categorizing human populations,

2) Define categories in as much detail as the study protocol allows,

3) Justify their choices of definitions and categories,

4) Explain whether (and if so, how) they controlled for confounding variables such as socioeconomic status, nutrition, environmental exposures, or similar factors in their analysis,

5) Update outmoded terms and potentially stigmatizing labels to more current, acceptable terminology. Examples: “Caucasian” should be changed to “white” or “of [Western] European descent” (as appropriate); “XXX victims” should be changed to “patients with XXX.

In this paper we used a national publicly available dataset and used its pre-defined categorization. Therefore, no method for categorizing human population was performed in this work. To address the issue of confounding variables, we eliminate highly correlated variables as explained in the “Pre-processing and feature selection” subsection. We have revised and changed the terms we used and these are in line with the current acceptable terminology.

5. Please ensure that you refer to Figure 4 in your text as, if accepted, production will need this reference to link the reader to the figure.

We have added text referring to this Figure in the Supporting Information section.

6. Please provide additional details regarding participant consent. In the Methods section, please ensure that you have specified (1) whether consent was informed and (2) what type you obtained (for instance, written or verbal). If your study included minors, state whether you obtained consent from parents or guardians. If the need for consent was waived by the ethics committee, please include this information.

We used a national publicly available dataset. Public use data sets are prepared with the intent of making them available for the public. The data available are not individually identifiable and therefore consent from our end was not required.

**Reviewer #1**

1. Introduction: update data of COVID cases in Brazil at revision's day

We have updated this information.

3.Discussion: discuss better the role of cardiovascolar risk factor on outcome (see and cite https://doi.org/10.1016/j.) and future perspective from your data (see and cite doi:10.3390/ijerph17082690)

Interesting connection of our results with <https://doi.org/10.1016/j> Thanks for pointing this out, we have reflected changes in the manuscript and added a sentence which uses <https://doi.org/10.1016/j> to support our results. As for the future perspective of our data, given the volatile and exponential nature of the spread of SARS-CoV-2019, we prefer not to predict future behavior for cases/testing in Brazil, nor other LMICs.

**Reviewer #2**

(...) The new information provided by this study is its association with demographics and the socioeconomic factors. However, authors have not put the details in the result section or discussion. The result section can be strengthen. Lots of data have been provided in the tables/ figures which are not mentioned in the result section. For better understanding of the manuscript by the readers, authors need to include these data in the result section.

We agree that we could improve the results section to highlight important variables and make it easier for the reader. We have updated the “Results” section incorporating relevant information from the tables to the text. Hopefully, these changes make our “Results” section to be more precise on the message we are delivering to readers. In general, using common practice, we have kept discussion in the “Results” to a minimum. More extensive discussion of key results is included in the “Discussion” section. Please also keep in mind that there are page/word limits.

Although mentioned in the tables and figures, authors need to discuss more about the educational level and access to private hospitals in the discussion section.

In the results section we have included the differences on the OR for patients with different schooling levels, as well as the OR for patients treated at public and private hospitals. Moreover, in paragraphs two and three (for schooling and care type) of the discussion section, we give context to the meaning of the variables and we highlight the disparities we observe in the results section. To make it clearer for readers, we have rephrased and added some information to these sentences.

Ultimate aim of the manuscript is to predict mortality and the need for mechanical ventilators for a COVID-19 patient using demographics, comorbidities, symptoms, and some clinical information. Authors should provide their suggestions about “how these parameters can be used by the healthcare professionals”.

We do not expect healthcare professionals to use this exact model. Rather, this work aims to open the discussion about socioeconomic disparities in health access, as well as, to help inform decisions on how to allocate medical resources and design targeted policies for vulnerable subgroups. This is stated in the “Study limitations” section of the manuscript as well as a new set of sentences we have added in the “Discussion” section.

One of the major causes for the mortality could be longer access to healthcare and late reporting of the case. Can authors provide any data (if possible) and mention it in the result/ discussion section.

Indeed, mortality could be correlated with late reporting of a case or longer hospitalization periods. In that sense, our dataset is more balanced than others as we only utilize observations of patients who have been hospitalized (the disease has already progressed for everybody in our study). In addition, we included a note in the “Study Limitations” section which acknowledged the possibility that the dataset is biased towards more critical cases (hospitalized patients). Unfortunately, the dataset at hand does not include information on how long was the stay of a patient at the hospital. Nevertheless, as mentioned in the previous comment, these models are developed to foster the discussion on socioeconomic/racial disparities and to suggest that the definition of vulnerable groups encompasses socioeconomic, health-related and demographic features, which should be used to design targeted policies.

Table 1: Why in the demographics section schooling and region are not equating to 100%

This is because there are missing values for that specific variable. We have included a sentence to address this misunderstanding. Thanks for bringing this up.

Line 214: check the sentence

We have changed and changed this sentence.
